# Prevalence of Human Papillomavirus Genotypes in Tehran, Iran

**DOI:** 10.34172/jrhs.2022.88

**Published:** 2022-10-19

**Authors:** Zahra Shalchimanesh, Maryam Ghane, Ebrahim Kalantar

**Affiliations:** ^1^Department of Biology, Islamshahr Branch, Islamic Azad University, Islamshahr, Iran; ^2^Department of Immunology, Faculty of Allied Health Sciences, Iran University of Medical Sciences, Tehran, Iran

**Keywords:** Co-infection, Genotype, Human papillomavirus

## Abstract

**Background: **Human papillomavirus (HPV) infection is a major cause of cervical cancer worldwide. Knowledge of the geographical distribution and epidemiology of the most common HPV genotypes is a crucial step in developing prevention strategies. Therefore, this study aimed to investigate HPV genotype distribution among HPV-positive women and men in Tehran, Iran.

**Study Design: **A case series study.

**Methods: **The study was performed on 219 HPV-positive individuals (160 females and 59 males) from Tehran, Iran. Samples were obtained from the cervix and vagina of female subjects and the genital warts of male subjects. DNA was extracted from samples, and a polymerase chain reaction (PCR)-reverse dot blot genotyping chip was used to examine HPV genotypes. Formalin-fixed, paraffin-embedded tissue samples of 51 patients from the study population were also included in this study.

**Results: **The proportion of high-risk (HR)-HPV was 67.12%. The most common HR-HPV types were HR-HPV16 (17.4%), HR-HPV68 (11.4%), and HR-HPV51 (7.8%). The most common low-risk (LR)-HPV types included LR-HPV6 (31.1%), LR-HPV81 (11.9%), and LR-HPV62 (11.4%). The highest prevalence of HPV was in the age group of>30 years (42.9%). Co-infection with multiple HR-HPV types was observed in 22.4% of specimens. Moreover, HR-HPV was found in 50% of women with normal cytology, 100% with a low-grade squamous intraepithelial lesion, and 84.61% with atypical squamous cells of undetermined significance.

**Conclusion:** The results indicated the remarkable growth of HR-HPV68, which has rarely been reported in Iran. The findings add knowledge to HPV epidemiological investigation and emphasize the need for introducing educational programs in high schools and appropriate vaccination in Iran.

## Background

 Human papillomavirus (HPV) is the most frequent cause of sexually transmitted infections in women and men. Epidemiological studies confirm a strong association between HPV and genital warts, cancer of the cervix, vagina, vulva, penis, and anus.^1,2^ The HPV infection has a worldwide distribution and is an established etiological factor for cervical cancer.^3^ More than 90% of cervical cancers and their precursor lesions are related to HPV infection.^4^ Various HPV genotypes can cause a variety of infections, including genital warts, common warts, low- and high-grade squamous intraepithelial lesions, recurrent respiratory papillomatosis, and cervical cancer. Cervical cancer is the second most frequent cancer among women worldwide and is responsible for at least 15% of all cancers among females in developing countries.^5^

 HPVs are grouped into mucosal and cutaneous types.^6^ Mucosal types can target the mucous membranes and cause anogenital warts in both children and adults and cervical neoplasia in adults only. Cutaneous types, on the other hand, target the squamous epithelium of the skin and cause common warts.^5^

 Among the 200 known HPV types, more than 40 have been identified in the anogenital region, which are further subdivided into low- and high-oncogenic risk.^7^ Low-risk (LR) types (LR-HPV6, 11, 34, 40, 42, 43, and 44) are most commonly associated with benign genital warts known clinically as condyloma but are also implicated in the development of laryngeal papilloma.^4,8^ So far, 15 HPV types have been identified as high-risk (HR), of which HR-HPV16 and HR-HPV18 are responsible for approximately 70% of all cervical cancer cases and are considered the most common HPV types worldwide.^1^ The possible oncogenic-risk or possible high-risk (pHR) types (26, 30, 34, 53, 66, 67, 69, 70, 73, 82, 85, and 97) are detected in a smaller proportion of cervical cancer, compared to HR-HPV16 and HR-HPV18.^4^ In a meta-analysis study conducted by Salavatiha et al in Iran, the most frequent HPV types were 16 (3.1%), 18 (1%), 6 (0.8%), 66 (0.8%), and 31 (0.7%) among women with normal cervix, and 16 (26%), 18 (7%), 6 (8%), 56 (7%), and 66 (7%) in women with atypical squamous cells of undetermined significance. They also reported HPV16 (53%), 18 (15%), 6 (6%), 11 (5%), and 31 (4%) as the most common HPV types among women with invasive cervical cancer.^9^

 Proper management of cervical cancer depends on early detection of the disease and effective prophylactic vaccines.^10^ The oncogenic nature of different types of HPV highlights the significance of the detection and typing of HPV isolates. There are currently three HPV vaccines available (Cervarix, Gardasil, and Gardasil 9), which are approved for use in many countries.^2,11^ However, knowledge of the HPV type distribution in each country is important for vaccine development and national vaccine planning. Regional data on the genotype distribution of HPV is necessary before making decisions about public health policies and predicting the impact of currently available vaccines or even preparing vaccines in each region. With this background, this study aimed to assess the distribution of HPV types among Iranian females and males and determine the dominant HR and LR genotypes using polymerase chain reaction (PCR)-reverse dot blot genotyping chip. The authors also surveyed the demographic characteristic data, in addition to the HPV genotype determination. Furthermore, the authors performed a pathological study and determined the distribution of HR genotypes in patients with normal and abnormal cytology.

## Methods

 This case series study was performed on 219 HPV-positive individuals (160 females and 59 males). Between August 2019 and April 2020, 570 patients who were referred to Gholhak Laboratory, Tehran, Iran, were tested for HPV infection, and HPV-positive individuals were included in the study.

 The sample size was calculated according to the data reported by other studies in Iran^12-14^ and measured with the following equation considering a 95% confidence interval with an absolute precision of ± 5%.^15^

 The inclusion criteria were all women or men positive for HPV who were referred to the laboratory and agreed to participate in the study. On the other hand, pregnant women and subjects with a history of HPV vaccination were excluded. All subjects voluntarily participated in the study, and their informed consent was obtained. They were interviewed for their age, marriage, menopause, occupation, level of education, pregnancy, contraception methods, and history of smoking. Sampling was performed by specialists and laboratory experts. Female specimens were collected from the vagina and cervix by sterile swaps and male specimens from the biopsy of genital warts. Formalin-fixed, paraffin-embedded tissue samples of 51 patients (38 females and 13 males) from the study population was also included in this study. This study was confirmed by the Ethics Committee of the Islamic Azad Tehran Medical Sciences University - Pharmacy and Pharmaceutical Branches Faculty (IR.IAU.PS.REC.1399.035).

 DNA extraction was performed by QIAamp DSP Virus Spin Kit (QIAGEN, Germany). HPV genotyping was performed using the Direct Flow Chip HPV Kit (Master Diagnóstica, Granada, Spain). Briefly, 4 μL of extracted DNA was added to 36 µL PCR reagents, including DNA Polymerase and Master Mix, and PCR was performed in a thermal cycler (Techne TC-412 Thermal Cycler, UK) under the following conditions: 98°C for 5 minutes, 5 cycles of 98°C for 5 seconds, 42°C for 5 seconds, and 72°C for 10 seconds; 45 cycles of 98°C for 5 seconds, 60°C for 5 seconds, and 72°C for 10 seconds, and a final extension at 72°C for 1 minute. The genotyping of amplicons was performed using the reverse dot blot hybridization method with specific DNA probes fixed in a chip with a nylon membrane (Flow-Chip technology) in a HybriSpot 12 (HS12, Vitro Group®) instrument. The signal released from the chip was taken and assessed by the Hybrisoft software (version 12). This PCR-based method is able to detect 19 HR and possible HR (16, 18, 31, 33, 35, 39, 45, 51, 52, 56, 58, 59, 68, 26, 66, 53, 69, 73, and 82), as well as 17 LR (6, 11, 40, 42, 43, 44, 55, 54, 61, 62, 67, 70, 71, 72, 81, 84, and 89) genotypes.

 Statistical analysis was performed using the chi-squared test. The SPSS software (version 24) was used to analyze the data. *P* < 0.05 was considered significant.

## Results

 The mean age of women and men (mean ± SD) was 32.03 ± 7.96 and 35.54 ± 7.49, respectively. The mean age of the total subjects was 32.97 ± 7.79.

 Based on the obtained results, the frequency of HR-HPV was higher than LR-HPV in the studied population. Out of 219 patients who participated in this study, 147 (67.12%) had HR genotypes, and 72 (32.9%) had LR genotypes.

**Figure 1:**
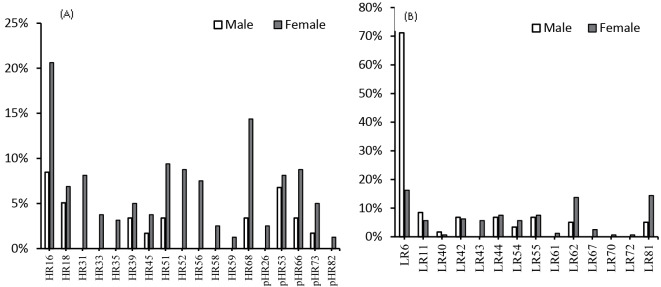
Distribution of High-risk, possible High-risk (A), and Low-risk Human papillomavirus genotypes among women and men

 The most frequent HR-HPV genotypes were 16 (17.4%), 68 (11.4%), and 51 (7.8%), and the most common LR genotypes were 6 (31.1%), 81 (11.9%), and 62 (11.4%) (Table 1). In the present study, single and multiple LR-HPV infections were observed in 71 (32.4%) and 58 (26.5%) patients, respectively. In the case of HR-HPV, 98 (32.9%) patients had a single type, and 49 (22.4%) patients had multiple HR-HPV infections. Mixed HR-HPV and LR-HPV infection was found in 60 (27.4%) patients. The distribution of HR- and LR-HPV genotypes in women and men are shown in Figure 1. The results showed that the highest frequency of HR genotypes in men and women was related to genotype 16. LR-HPV6 was also the most common LR genotype among men and women. The relationship between demographic characteristics and the two HPV infection statuses (HR and LR) of the study population are presented in Table 2. According to the results, a significant relationship was observed between age and infection status (*P* < 0.001), and the prevalence of HR-HPV was higher than LR-HPV in the age group of 18-30 years. The findings also revealed that gender was significantly related to infection status (*P* < 0.001). The prevalence of HR-HPV, compared to LR-HPV, was higher in women. The number of sexual partners was also related to infection status (*P*= 0.028). The rate of HR-HPV increased significantly with an increase in the number of sexual partners, had HR-HPV. There was a significant relationship between menopause and infection status (*P* < 0.001). On the other hand, variables such as education, smoking, occupation, husband and wife being away, marriage, and warts were not related to HPV infection statuses.

**Table 1:** Distribution of human papillomavirus genotypes in the study population (n = 219)

**Genotypes**	**Number**	**Percent**
HR		
HR16	38	17.4
HR18	14	6.4
HR31	14	6.4
HR33	6	2.7
HR35	5	2.3
HR39	10	4.6
HR5	7	3.2
HR51	17	7.8
HR52	14	6.4
HR6	12	5.5
HR58	4	1.8
HR59	2	0.9
HR68	25	11.4
PHR		
pHR26	4	1.8
pHR53	17	7.8
pHR66	16	7.3
pHR73	9	4.1
pHR82	2	0.9
LR		
LR6	68	31.1
LR11	14	6.4
LR40	2	0.9
LR42	14	6.4
LR43	9	4.1
LR44	16	7.3
LR54	11	5.0
LR55	16	7.3
LR61	2	0.9
LR62	25	11.4
LR67	4	1.8
LR70	1	0.5
LR72	1	0.5
LR81	26	11.9

HR: High-risk; PHR: Possible high-risk; LR: Low-risk.

**Table 2:** Demographic characteristics and the two HPV infection statuses (high-risk and low-risk) of the study population (n = 219)

**Variables**	**Total**	**High-risk, n=147**		**Low-risk, n=72**		***P*** ** value**
		**Number**	**Percent**	**Number**	**Percent**	
Age group (y)						0.001
18-30	94	76	80.8	18	19.1	
30-40	89	49	55.1	40	44.9	
40-50	31	20	64.5	11	35.5	
50-60	5	2	40.0	3	60.0	
Sex						0.001
Male	59	19	32.2	40	67.8	
Female	160	128	80.0	32	20.0	
Education						0.527
Completed school leaving certificate	68	43	63.2	25	36.8	
Illiterate/Primary/Secondary	100	71	71.0	29	29.0	
Higher degree	51	33	64.7	18	35.3	
Occupation						0.249
Yes	88	63	71.6	25	28.4	
No	131	84	64.1	47	35.9	
Full term pregnancy						0.021
0	30	19	63.3	11	36.7	
1	52	41	78.8	11	21.2	
> 1	78	68	87.2	10	12.8	
Husband/Wife away from home						0.267
Yes	77	48	62.3	29	37.7	
No	142	99	69.7	43	30.3	
Smoker						0.582
Yes	44	28	63.6	16	36.4	
No	175	119	68.0	56	32.0	
Number of life sex partners						0.028
0	29	15	51.7	14	48.3	
1	136	89	65.4	47	34.6	
> 1	54	43	79.6	11	20.4	
Menopause						0.001
Pre	100	89	89.0	11	11.0	
Post	60	39	65.0	21	35.0	
Marital status						0.174
Divorced	50	39	78.0	11	22.0	
Married, the husband has only one wife	129	82	63.6	47	36.4	
Married, the husband has another/other wife/wives	40	26	65.0	14	35.0	
Wart						0.978
Yes	94	63	67.0	31	33.0	
No	125	84	67.2	41	32.8	
Contraception use						0.478
Yes	75	48	64.0	27	36.0	
No	144	99	68.8	45	31.3	

**Figure 2:**
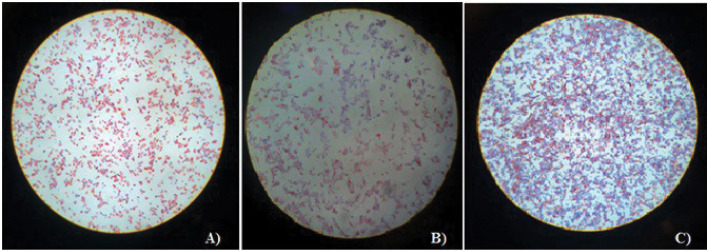
Representative images of cervical specimens. A) Normal tissue; B) Low-grade squamous intraepithelial lesion cells; C) Atypical squamous cells of undetermined significance cells (hematoxylin and eosin stain, × 200)

 Out of 219 patients studied, 51 (38 females and 13 males) had pathological specimens. The clinical diagnoses included 35 (66%) cases of normal tissues, 13 (25%) cases of atypical squamous cells of undetermined significance (ASC-US), and 5 (9%) cases of Low-grade squamous intraepithelial lesion (LSIL). The representative images of the cervical specimen are shown in Figure 2. In the present study, all men had normal cytology, and 23% were infected with HR-HPV. In women, HR-HPV was found in 10 (50%) with normal cytology, 5 (100%) with LSIL, and 11 (84.61%) with ASC-US cells.

## Discussion

 Studies show that there is a significant association between persistent infection of HR types of HPV and cervical cancer.^16^ The reports of HPV genotyping in cervical cancer indicate that the distribution of HPV genotypes varies depending on race and geographical region.^17^ Therefore, epidemiological data on HPV genotypes in each country can be very useful for implementing appropriate strategies to prevent cervical cancer. This study provides a recent report regarding HPV genotype distribution among women and men in Tehran, Iran, using an accurate molecular method.

 Based on the results, 67.12% and 32.9% of the patients had HR- and LR-HPV genotypes, respectively. Similar to the present findings, in a study by Chalabiani et al, the prevalence of HR-HPV in different parts of Iran was 67.2%.^18^ In this study, the highest age group of women with HPV infection was < 30 years old, and most women who had both LR and HR genotypes at the same time were also < 30 years. These findings underscore the need to introduce educational programs in high schools to raise awareness about HPV infection. In a study by Taghizadeh et al, the highest age group of affected women was reported to be between 30-40 years,^19^ whereas, in many countries, the highest prevalence of this virus is typically observed in lower age groups, which is justified by factors such as age at the onset of sexual activity, marriage age, unusual sexual behavior and activity, culture, economic conditions, and smoking.^20^ As an example, in the report of Coser et al, the high prevalence of HPV infection and its persistence in women under 30 years have been observed in a region of Brazil which also suffered from financial poverty.^21^ In the present results, as in some studies, the rate of infection has decreased gradually with aging.^1,22^

 In the present study, a total of 32 HPV genotypes were identified, including 18 HR and possible HR types (HPV16, 18, 26, 31, 33, 35, 39, 45, 51, 52, 53, 46, 58, 59, 66, 68, 73, and 82), as well as 14 LR types (HPV6, 11, 40, 42, 43, 44, 54, 55, 61, 62, 67, 70, 72, and 81). Among the LR types, HR-HPV6 was higher than other types, which indicates the frequency of this genotype in Iran. In many studies conducted in different parts of Iran and the world, the same result has been achieved.^18,22^

 Consistent with the findings of other studies,^2,23,24^ this study reported HR-HPV16 as the most prevalent HR-HPV type in the study population. HR-HPV16 has the greatest potential for carcinogenesis and is the key candidate for routine HPV vaccines.^25^ In some studies conducted in Iran and other countries, HR-HPV18 has been reported as the second most common genotype after HR-HPV16.^19,26,27^ Contrary to their results, the present study found HPV68 as the second most common genotype. In other studies, genotypes 31, 35, 39, 53, and 58 have been reported, in addition to genotype 16.^2,18,23,24,28^ These differences in the HPV genotype distribution indicate the divergence of HPV prevalence in differing areas.^29^

 The results showed that among patients with both LR and HR genotypes, the frequency of HR-HPV16, 68, and 51 types, as well as LR-HPV6 and 81 types, was higher than other genotypes. In the present study, single and multiple HR-HPV infections were observed in 32.9% and 22.4% of patients, respectively. The rate of multiple HPV infections obtained in this study is higher than previous reports from China (20.37%) and Italy (10%).^2,30^ A higher prevalence was also reported in Iran (28.4%)^13^ and Mexico (67.3%).^31^ The genotypes achieved in the present study can reflect the frequent exposure of these patients to different types of HPV due to unprotected sexual activity and having multiple sexual partners. Therefore, social investigation of this issue can help social health and strengthen the family.

 In the present study, among women, HR-HPVs were found in 10 (50%) with normal cytology, 5 (100%) with LSIL, and 11 (84.61%) with ASC-US cells. The findings are consistent with those of the study by Rogovskaya et al, based on data from 12 countries. They found that the prevalence of HR-HPV in women with normal cytology was in the range of 0.0% to 48.4%, with low-grade cervical lesions in the range of 29.2% to 100%, and with high-grade cervical lesions in the range of 77.2% to 100%.^32^ It has been reported that the prevalence of HR-HPV genotypes increases with the grade of cytological lesions.^33^ However, in the present study, HR-HPV was more prevalent in LSIL (100%) than ASC-US (84.61%), which may be due to the low number of LSIL and ASC-US cases included in the present study.

 The limitations of the present study should be considered. The study lacked enough cytological information to examine its correlation with genotyping results. Moreover, due to the sample size limitation, the prevalence of HPV genotypes obtained in this study may not reflect the distribution of HPV genotypes in the country. Therefore, larger studies are needed to confirm the obtained results.

 The strengths of this study include variation in patients’ age, diversity of samples, and using the reverse dot blot hybridization chip molecular method to determine the genotype of the virus. The use of such molecular methods leads to more accurate diagnosis and preventive measures to prevent the progression of primary cellular changes to cervix neoplasia and can be an effective step in controlling and treating dominant genotypes.

**Highlights**

- Co-infection with multiple human papillomavirus (HPV) genotypes was common in the HPV-positive population under study.
- The highest prevalence of HPV was in the age group under 30 years.
- A remarkable growth of high-risk (HR)-HPV68 was observed, which has rarely been reported in Iran.
- The rate of HR-HPV increased significantly with an increase in the number of sexual partners.

## Conclusion

 The findings showed that co-infection with multiple HPV genotypes is common in the HPV-positive study population, and HPV16 was reported as the most predominant genotype among patients. The study also reported remarkable growth of HPV68, which has rarely been reported in Iran.These data may be helpful for vaccine development and national vaccine planning.

## Acknowledgments

 The authors wish to thank the staff of Gholhak Laboratory for their assistance. This article was extracted from an MSc student thesis.

## Conﬂict of interest

 The authors declare that they have no potential conflict of interest related to this study.

## Funding

 None.
